# A Theory-Based Video Messaging Mobile Phone Intervention for Smoking Cessation: Randomized Controlled Trial

**DOI:** 10.2196/jmir.1553

**Published:** 2011-01-21

**Authors:** Robyn Whittaker, Enid Dorey, Dale Bramley, Chris Bullen, Simon Denny, C Raina Elley, Ralph Maddison, Hayden McRobbie, Varsha Parag, Anthony Rodgers, Penny Salmon

**Affiliations:** ^7^The Quit GroupWellingtonNew Zealand; ^6^The George Institute for International HealthSydneyAustralia; ^5^Queen Mary University of LondonLondonUnited Kingdom; ^4^Department of General Practice & Primary Health CareUniversity of AucklandAucklandNew Zealand; ^3^Department of Community PediatricsUniversity of AucklandAucklandNew Zealand; ^2^Waitemata District Health BoardAucklandNew Zealand; ^1^Clinical Trials Research UnitUniversity of AucklandAucklandNew Zealand

**Keywords:** smoking cessation, cellular phone

## Abstract

**Background:**

Advances in technology allowed the development of a novel smoking cessation program delivered by video messages sent to mobile phones. This social cognitive theory-based intervention (called “STUB IT”) used observational learning via short video diary messages from role models going through the quitting process to teach behavioral change techniques.

**Objective:**

The objective of our study was to assess the effectiveness of a multimedia mobile phone intervention for smoking cessation.

**Methods:**

A randomized controlled trial was conducted with 6-month follow-up. Participants had to be 16 years of age or over, be current daily smokers, be ready to quit, and have a video message-capable phone. Recruitment targeted younger adults predominantly through radio and online advertising. Registration and data collection were completed online, prompted by text messages. The intervention group received an automated package of video and text messages over 6 months that was tailored to self-selected quit date, role model, and timing of messages. Extra messages were available on demand to beat cravings and address lapses. The control group also set a quit date and received a general health video message sent to their phone every 2 weeks.

**Results:**

The target sample size was not achieved due to difficulty recruiting young adult quitters. Of the 226 randomized participants, 47% (107/226) were female and 24% (54/226) were Maori (indigenous population of New Zealand). Their mean age was 27 years (SD 8.7), and there was a high level of nicotine addiction. Continuous abstinence at 6 months was 26.4% (29/110) in the intervention group and 27.6% (32/116) in the control group (*P* = .8). Feedback from participants indicated that the support provided by the video role models was important and appreciated.

**Conclusions:**

This study was not able to demonstrate a statistically significant effect of the complex video messaging mobile phone intervention compared with simple general health video messages via mobile phone. However, there was sufficient positive feedback about the ease of use of this novel intervention, and the support obtained by observing the role model video messages, to warrant further investigation.

**Trial registration:**

Australian New Zealand Clinical Trials Registry Number: ACTRN12606000476538; http://www.anzctr.org.au/trial_view.aspx?ID=81688 (Archived by WebCite at http://www.webcitation.org/5umMU4sZi)

## Introduction 

While smoking prevalence has been declining in many countries [1,2] high prevalence rates are a cause for concern in developing countries [3-5], in disadvantaged or vulnerable populations [6-8], and in young people [8-10]. New Zealand Maori (the indigenous population of New Zealand) have particularly high smoking prevalence rates (40.4% of males and 49.7% of females aged 15-64 years [10]) and new interventions must be appropriate for this population group in New Zealand.

Smoking quit rates are low even where intensive behavioral and pharmacological support is available [11], although most smokers who try to quit do so without extra assistance [12]. Providing more options for smoking cessation support is one strategy to try to encourage more quit attempts. Mobile phones have good potential as one option because they tend to be always with people, and messages can be sent directly to quitters wherever they are and at the most appropriate times (eg, for cravings or for usual cues to smoke). There is some evidence of more equitable access to mobile phones than to other communications services in developed countries [13,14] and rapid uptake in developing countries [15,16]. There is also emerging evidence that those with high health needs may use mobile phones more than those without [13,17,18].

Our successful text messaging smoking cessation program [19] was recently implemented as a national government-funded program in New Zealand [20]. In order to use new advances in mobile phone technology to continue to improve uptake and effectiveness, we proposed and developed an updated intervention (“STUB IT”).

A randomized controlled trial was undertaken between November 2007 and August 2009 to determine whether a video-based smoking cessation intervention delivered via mobile phone was effective at increasing smoking cessation rates compared with a control group over a 6-month period. In this paper we describe this trial, and reflect on challenges faced in recruitment that undermined its capacity to adequately test the effectiveness of the intervention.

## Methods

Recruitment was targeted at young adults (16-25 years) and particularly toward young Maori. The study was advertised extensively via radio, internet, mobile phone (to those who had signed up for such a service), paper-based and online magazines, Maori-specific media of all types, local and national newspapers, and media releases to national media outlets. Advertisements were placed in tertiary education institutions (via campus posters, student magazines, student websites, student health services, and student radio), primary health care services, smoking cessation services, large employer health promotion programs, and posters or leaflets at cafes, bars, and sports grounds.

**Figure 1:**
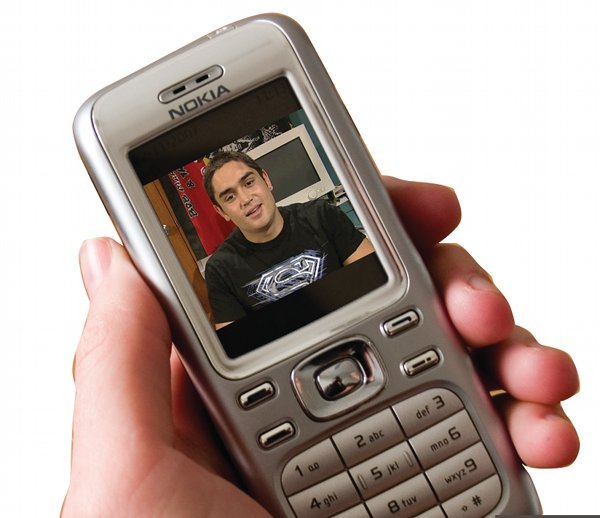
Screenshot of the intervention

Participants were eligible if they were at least 16 years of age, smoked daily, and wanted to quit. Participants were required to have a mobile phone that was capable of receiving video messages. The video messages were sent as a text message with a universal resource locator (URL) address in the text. Participants highlighted the URL to trigger automatic downloading and playing of the video on the phone (Figure 1; see also Multimedia Appendix 1 and 2 for sample videos). Participants could return to the text message to replay the video if desired. This process does not require extremely high-end technology phones, but was available on most recent mobile phones. The video messages were made as small as possible (<300 kB) to allow the lowest-specification common phone to be able to access them. Due to a partnership with Vodafone New Zealand Ltd (one of only two mobile phone networks in New Zealand at the time), this whole process was free to participants. 

Potential participants completed an online eligibility check and, if eligible, were advised to read the study information online (or it could be emailed or posted) and reply to a consent text message with the words “I consent.” Consenting participants were directed to complete baseline data collection on an online form. On submission of this information, computer randomization allocated participants to an intervention or control group, using stratified minimization for age (25 years and under, over 25 years), ethnicity (Maori, non-Maori), and level of nicotine dependence (time to first cigarette 30 minutes or less, more than 30 minutes).

Both groups nominated a quit day (QD) on which they aimed to stop smoking that was between 1 and 3 weeks from randomization. Participants also nominated two time periods (in a 24-hour clock) during which they wished to receive the mobile phone messages.

Those in the intervention group were also directed to an online brief description and photograph of the six role models (three of whom were Maori) and asked to select one person from whom they would receive messages (although they were able to change this later if desired). The steps in the development of the intervention have been described in detail elsewhere [21]. In brief, we drew on social cognitive theory [22] to inform the use of role models via short video messages providing observational learning. We hypothesized that this role modeling by “ordinary” young people would enhance self-efficacy to quit smoking and thereby increase the chances of a quit attempt being successful [23-25]. The video messages were filmed as video diaries during a quit attempt, with the role models discussing issues they had found difficult and the techniques and coping strategies they used to remain smoke-free. These vignettes were based on the role model’s own story (all six role models were ex-smokers), plus theory and evidence-based behavior change techniques usually taught in cessation counseling (such as setting goals, being reminded of reasons for quitting, identifying triggers and cues to smoking, planning to manage or avoid triggers and cues, receiving positive reinforcement, and using social support).

The intervention was arranged into a chronological schedule of mobile phone messages that included the role model videos, text messages (short message service; SMS), and other video messages (animations about reasons to stop smoking; and “truth” campaign mass media advertisements supplied by the American Centers for Disease Control and Prevention). Table 1 shows the number and type of messages in each phase, along with the duration of each phase.

**Table 1:** Chronological sequence of mobile phone messages

Phase	Number of messages	Timing and duration of phase	Format of messages in each phase
Countdown to QD^a^	1/day	For 1 week prior to QD	Role model videos and texts
QD	3/day	1 day (QD)	Role model videos and texts
Intensive phase	3/day	For 4 weeks post-QD	Role model videos and texts
Maintenance phase	1 every 2 days	For 2 weeks after intensive phase	SMS^b^ messages, other mixed videos
Maintenance continued	1 every 4 days	For about 20 weeks until 6 months after randomization	SMS messages, other mixed videos

^a^ Quit day.

^b^ Short message service, or text messages.

Additional features included a website for intervention group participants that allowed them to review video messages they had been sent (and rate them if desired), change their selected time periods, and change (or add to) their selected role model. Intervention group participants could also ask for extra support messages on demand by texting keywords to the study shortcode (four-digit number). Texting “crave” and the context (either “stress,” “bored,” or “drinking” – three common triggers for smoking in young adults) would result in the immediate automated sending of an appropriate video or text message on how to beat cravings within that context. Texting “relapse” would result in three messages over the next 90 minutes to motivate to keep going with the quit attempt and suggest ways of getting extra support. The control group participants received one video message every 2 weeks with general health messages and reminders about the study for 6 months. 

The primary outcome for the study was continuous abstinence as defined by the Russell standard [26], which allows up to five cigarettes over 6 months after QD. Other outcomes were 7-day point prevalence abstinence; confidence in ability to quit/stay quit (as a percentage on a scale from 0%, not confident, to 100%, fully confident); number of quit attempts and use of nicotine replacement therapies during the study period; participant satisfaction with aspects of the program (intervention group only); and any motor vehicle accidents that occurred while driving and using a mobile phone during the study period (as possible adverse events).

Smoking status was verified on a random sample of 10% of eligible participants prior to randomization. Verification of quitting status was attempted in all participants reporting continuous abstinence at 6 months using salivary cotinine reading on a mailed-out and returned NicAlert (Nymox Pharmaceutical Corporation, Hasbrouck Heights, NJ, USA) test-strip pack. Salivary cotinine has a half-life of 15-40 hours and is able to distinguish smokers from nonsmokers using a cutoff of 10 ng/mL of cotinine (sensitivity 93%, specificity 95%, and a positive predictive value of 95%) [27]. Two staff members independently read the NicAlert test strips.

The nature of the intervention ensured the study could only be single blinded – that is, participants were aware of which group they were allocated to. However, most data were collected via web-based forms completed by participants, and researchers involved in data collection, particularly outcome assessment, were blind to allocation.

Initial calculations indicated that a target sample size of 1300 participants would detect a relative risk of 1.75 for a control group 6-month quit rate of 8.5% (intervention group quit rate of 15%), with 90% power at *P* = .05. This included a loss to follow-up of 20%. All statistical analyses were performed using SAS version 9.1.3 (SAS Institute Inc, Cary, NC, USA), all statistical tests were two-tailed, and a 5% significance level was maintained throughout the analyses. The main analyses were based on the intention-to-treat principle as recommended for cessation studies [26], where participants lost to follow-up were considered not to have quit at follow-up. Simple chi-square analyses compared the proportion quit at different stages of follow-up between the intervention groups. 

## Results 

**Figure 2:**
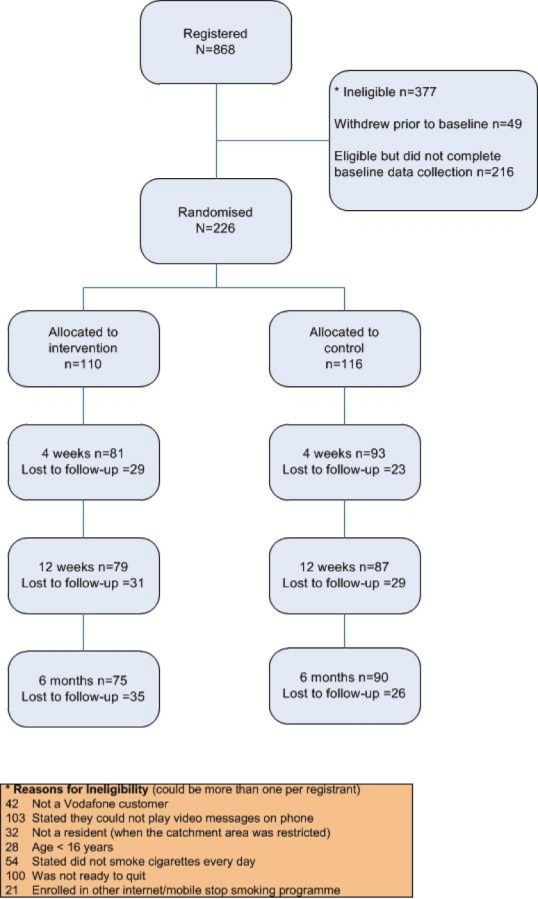
Consort flowchart for the randomized controlled study of STUB IT

Participants were recruited into the study between November 2007 and February 2009, and this proved much more difficult than expected. We attempted multiple sequential “waves” of recruitment efforts via new and multiple sources. However, each wave did little to change the overall recruitment rate. The study catchment area was also increased sequentially from the Auckland region (population approximately 1.4 million), to the Northern Region of the North Island (population approximately 2.3 million), to the whole of New Zealand (population 4.1 million). Initial incentives of monthly prize draws of new third-generation (3G) phones were deemed insufficient to attract new participants, and reimbursements to all participants for their time and participation were later added. Due to these problems, and the costs involved in recruitment, we decided to close the study to recruitment with 226 randomized participants. Figure 2 shows the numbers of registrants, randomized participants, and those completing follow-up. Due to the nature of online data collection at follow-up points, it was possible for participants to enter some follow-up data but not complete the entire form. The follow-up numbers presented in the figure are based on those providing the primary outcome data (at 6 months) and the main smoking outcomes data (at 4 and 12 weeks).

Table 2 shows the baseline characteristics of randomized participants. Due to the targeted recruitment strategies, the mean age of participants was 27 years; although there was no upper age limit and the oldest person in the study was 63 years old. The majority of participants were of New Zealand European ethnicity, with nearly 24% (54/226) of participants self-selecting Maori ethnicity. Baseline smoking characteristics were similar in the two groups, with some indication that this was a highly addicted cohort due to Hooked on Nicotine Checklist mean scores of 8 (SD 1.9) out of 10 [28].

**Table 2:** Baseline characteristics of randomized participants, n (%)^a^

		Intervention (n = 110)	Control (n = 116)
Mean (SD) age, years		27.5 (9.5)	26.6 (7.8)
Female		58 (52.7)	49 (42.2)
Ethnicity			
	New Zealand European	55 (50.0)	63 (54.3)
	Maori	24 (21.8)	30 (25.9)
	Pacific	12 (10.9)	5 (4.3)
	Asian	10 (9.1)	13 (11.2)
	Other	6 (5.5)	5 (4.3)
	Missing	3 (2.7)	0 (0)
Total income in previous 12 months			
	Less than NZ$30,000	53 (48.2)	51 (44.0)
	NZ$30,001-60,000	35 (31.8)	40 (34.5)
	Over NZ$60,000	7 (6.4)	12 (10.3)
	Don’t wish to answer	15 (13.6)	13 (11.2)
How soon after waking do you smoke?			
	Within 5 minutes	26 (23.6)	27 (23.3)
	6-30 minutes	45 (40.9)	52 (44.8)
	31-60 minutes	21 (19.1)	24 (20.7)
	After 60 minutes	18 (16.4)	13 (11.2)
Have you ever tried to quit smoking but couldn’t? Yes		102 (92.7)	104 (89.7)
Do you smoke now because it is really hard to quit? Yes		75 (68.2)	82 (70.7)
Have you ever felt addicted to tobacco? Yes		98 (89.1)	107 (92.2)
Mean (SD) Hooked on Nicotine Checklist (HONC) score		7.99 (2.11)	8.03 (1.68)
Mean (SD) confidence in being able to quit this time %		62.4 (22.0)	66.5 (21.8)

^a^ Unless otherwise stated.

Table 3 reports continuous abstinence rates (the primary outcome). Intention-to-treat continuous abstinence at 6 months was 26.4% (29/110) in the intervention group and 27.6% (32/116 in the control group (*P* = .8). Of the 61 participants reporting continuous abstinence at 6 months, 10 were either noncontactable or stated they had relapsed since the end of the study period (when they had claimed to have quit) and therefore could not undergo verification of quitting status. The remaining 51 were sent NicAlert test-strip packs and were contacted repeatedly to return the strips. Fourteen quitters in the intervention group (48% of 29) returned the strip and seven (24%) were confirmed as nonsmokers. Fifteen quitters in the control group (47% of 32) returned the strip and 11 (31%) were confirmed as nonsmokers.

**Table 3:** Continuous abstinence from quit day to 6 months, n (%)

Have you smoked tobacco at all since quit day?		Intervention	Control	*P*-value^a^
**Responders-only analysis**				.7
	Not a single puff or between 1 and 5 cigarettes	29 (38.7)	32 (35.6)	
	More than 5 cigarettes	46 (61.3)	58 (64.4)	
	Missing data	35	26	
**Intention-to-treat analysis**				. 8
	Not a single puff or between 1 and 5 cigarettes	29 (26.4)	32 (27.6)	
	More than 5 cigarettes or missing data	81 (73.6)	84 (72.4)	

^a^ *P*-value for chi-square test comparing groups.

No significant difference was found between the groups in the intention-to-treat point prevalence abstinence (no smoking at all in the past 7 days), which was recorded at three time points and is shown in Table 4.

**Table 4:** Point prevalence abstinence at 4 weeks, 12 weeks, and 6 months, n (%)

Have you smoked at all in the past 7 days?		Intervention	Control	*P*-value^a^
**4 weeks**				.8
	Not a single puff	12 (10.9)	14 (12.1)	
	Yes or missing data	98 (89.1)	102 (87.9)	
**12 weeks**				.3
	Not a single puff	30 (27.3)	25 (21.6)	
	Yes or missing data	80 (72.7)	91 (78.4)	
**6 months**				.99
	Not a single puff	25 (22.7)	26 (22.4)	
	Yes or missing data	85 (77.3)	88 (77.6)	

^a^ *P*-value for chi-square test comparing groups.

At 6 months those who reported quitting were asked to rate their confidence in being able to stay quit, and those who had relapsed were asked to rate their confidence in being able to quit again. There were no significant differences between the intervention and control group mean scores at any of these points (data not shown). 

At 6 months all participants were asked how many quit attempts they had made during the study period. In the intervention group 7/73 respondents (9.6%) and in the control group 4/81 (4.9%) (*P* = .3) stated they did not attempt to quit at all, but the majority of respondents in both groups made multiple quit attempts. In the intervention group 17 of 69 respondents (25%) and in the control group 26 of 68 respondents (38%) (*P* = .2) had used pharmacological quitting support (nicotine patches, nicotine gum, or nortryptiline) at any stage in the 6-month study period.

Participants in the intervention group were asked for their feedback on the program. In general the majority of responders stated they liked the video messages from quitters, and appeared to appreciate the frequency and timing of messages (Table 5, 6).

**Table 5:** Intervention group satisfaction with the program, n=67 (%)^a^

Which aspects did you…	like?	dislike?	no comment	did not use
That I would relate to quitters	46 (69)	3 (4)	13 (19)	5 (7)
What quitter has to say	44 (66)	6 (9)	10 (15)	7 (10)
Video messages from quitters	43 (64)	6 (9)	9 (13)	9 (13)
The timing of messages	41 (61)	15 (22)	10 (15)	1 (1)
Receiving lots of messages	39 (58)	20 (30)	6 (9)	2 (3)
The website	34 (51)	3 (4)	16 (24)	14 (21)
Crave messages	32 (48)	8 (12)	8 (12)	19 (28)
Antitobacco industry messages	25 (37)	13 (19)	10 (15)	19 (28)
Animations	23 (34)	3 (4)	8 (12)	33 (49)

^a^ Missing data have been excluded.

**Table 6:** Aspects of the program that aided cessation in the intervention group

Which aspects helped you to stop smoking even if you relapsed later?	Yes
Watching someone like me go through the quitting process	59 (88)
Being supported to feel like I could do it	55 (86)
Feeling like I belonged/like others were going through same thing	52 (81)
Things the people in the video clips said	50 (76)
Getting messages at the right times	47 (75)
The free stuff	44 (69)
It was fun	39 (61)
Made me get support from my friends or family	39 (60)
The website/other people videos	35 (57)
Realizing I had been manipulated by tobacco industry	31 (48)
Messages/games/whatever distracting me from cravings	30 (47)
Crave messages	29 (45)

Free text answers to what they liked most about the program could be divided into three groups: those who reported something about feeling supported (29/54, eg, from the role model, because they felt part of a group, because others were going through it too); those whose comments related to the program (11/54, eg, timing of messages, constant messages, nonintrusiveness, use of technology); and those who said all of it (5/59).

When asked what they disliked most about the program, 20/49 said they disliked nothing, six complained of some sort of technical issue, and seven did not feel the content was right or did not relate to the models. Five said there were too many messages, one said the messages reminded them to smoke, and one had the (false) perception they were being charged for messages. The most common suggestions to improve the program were around having more personal (human) contact, individually or via support groups or internet social networking.

A report from the intervention program confirmed that 29/110 participants (26.4%) had used the text “crave” function and 18/110 (16.4%) the text “relapse” function.

Equal numbers of participants in each group (n = 4) reported having a motor vehicle accident during the study period where the participant was the driver. In the control group one such accident occurred while the participant was using their mobile phone, one within 5 minutes of receiving a message, and two while the participant was smoking, whereas none of the accidents in the intervention group were reported as being temporally related to mobile phone use or smoking.

## Discussion 

This study is the first to have developed and trialed a smoking cessation intervention delivered via video messaging on mobile phones. We found no significant differences in quit rates between the intervention and control groups (with trends in different directions depending on time point and type of analysis). However, the trial was substantially underpowered due to our failure to recruit sufficient participants to reach the desired sample size and the higher than expected self-reported control group quit rate. In fact, quit rates in both groups were high compared to New Zealand’s quitline quit rates of 17% (6-month continuous abstinence) and 10% in the 18- to 24-year age group, but similar to those reported in our previous study of a text messaging cessation intervention [19]. Therefore, it is possible that with adequate power, an effect may have been found.

The strengths of the study include a study design in accordance with CONSORT guidelines and the strict definitions and analysis of smoking abstinence outcomes. We also used theory on which to base the intervention: this has been shown to be important in technology-based health behavior change [29] and ensures the intentions and drivers in the development of the intervention are clear and replicable. Indeed, participants commented positively on the use of role models as a means of support in their quitting attempts.

The obvious limitation of the study is the suboptimal recruitment. There are several potential reasons for this, which present challenges to be addressed in future trials. First, our recruitment efforts were targeted at adolescents (16 years and over) and young adults. We found that, despite indicating their interest in quitting, most young people were not actually ready to commit to a cessation intervention. This has been demonstrated elsewhere in focus groups and surveys of young people [30-32]. The recent updated Cochrane review of smoking cessation interventions for young people [33] commented that many of the included studies were underpowered, with only 5032 participants from 24 studies. Only two of these studies recruited directly from the community as we did. Lipkus and colleagues randomized 402 participants despite approaching nearly 40,000 young people in shopping malls [34], while Patten et al required 42 months to randomize 139 participants [35]. Recruitment to youth smoking cessation services has also been shown to be problematic [36], as has recruitment of youth to other types of research [37].

Second, the costs of messaging and advanced technology may have proved a barrier for some. At the time of recruitment, New Zealand mobile phone data charges (or anything other than SMS and voice calling) were expensive. We spoke to two participants who were wary of being charged (despite being advised the program was free) and there may have been more who did not register for this reason. Also many people were unaware whether their mobile phone could receive video messages. These factors may have dissuaded people even registering their interest and therefore we have no information on their relative importance in our recruitment. However, if poor recruitment was related to a wariness of new multimedia messaging, we feel that this will have been short-lived: in our current trial of a multimedia mobile phone program to prevent adolescent depression we have recruited 1200 participants over 30 school weeks.

Thirdly, plans to incentivize participation were hampered by several factors. Monetary incentives are considered to be effective in encouraging participation of adolescents in research [38], so we planned to offer free data or top-ups to participants’ mobile phone accounts. After commencing the study this was deemed not technically possible, so instead we instituted monthly prize draws of new 3G mobile phones. However, the ethics committee did not approve promotional material that advertised the prize draws. Nevertheless, when recruitment was found to be falling behind target, we obtained ethics approval to provide participants with vouchers (for a mobile phone, the supermarket, or gasoline) as reimbursements for their time, and recruitment rose in response but was not sufficient to make a large difference.

Finally, and somewhat ironically, the text messaging cessation program trialed in our own earlier study [19] may have provided competition with our trial: the *tx2quit* program went live in New Zealand in June 2008 with national promotion by Quitline, and recruited approximately 4000 participants in the following 12 months [20]. 

Mobile phones are increasingly being used globally in health services as a means of more frequent and convenient contacts with health providers [39,40], remote monitoring of progress [41,42], and to reduce wastage of scarce health resources [40,43]. There are several aspects of mobile phones that also make them a valuable component of healthy behavior change support, such as being with people in times of need, providing two-way communications for help on demand, allowing proactive reminders of motivations to change behavior, providing social support from people’s own networks, and providing a long-term means of support [44]. A Cochrane systematic review of the use of mobile phones in smoking cessation support programs [45] demonstrated short-term effectiveness of mobile phone-only programs and long-term effectiveness of a mobile phone and internet program.

This study adds to this body of knowledge by demonstrating the feasibility and participant appreciation of video messages via mobile phones to provide observational learning and support for healthy behavior change. It is also of note that participants were happy to complete research procedures such as consent and data collection by mobile phone. Indeed we achieved higher response rates to text message questions (217/226 or 96% response rate to a question about confidence at QD and 170/226 or 75% response rate to a smoking status question at 12 weeks post-QD) than to our online data collection forms (despite text message reminders to complete them).  

In conclusion, this trial struggled to recruit participants, in particular young adults who wanted to quit smoking. This may explain the failure to show an effect of the intervention, or it may be that the complex theory-based intervention is no more effective than simple less-frequent video messages from researchers. However, there was sufficient positive feedback about the support obtained by observing the role models in the program to warrant further investigation in this area. Further research should explore the effect of this role model-based mobile phone smoking cessation intervention for older adults – a group that are perhaps more serious about stopping smoking and are becoming higher users of newer mobile phone technology.

## Multimedia Appendix 1

Example of one of the role model video messages from the STUB IT intervention

## Multimedia Appendix 2

Second example from another role model of their last video message in the intervention program

## Abbreviations

3G: third-generation mobile phone network
QD: quit day
SMS: short message service/text messages
URL: universal resource locator

## References

[1] Centers for Disease Control and Prevention (CDC) (2009). State-specific prevalence and trends in adult cigarette smoking--United States, 1998-2007. MMWR Morb Mortal Wkly Rep.

[2] White V, Hill D, Siahpush M, Bobevski I (2003). How has the prevalence of cigarette smoking changed among Australian adults? Trends in smoking prevalence between 1980 and 2001. Tob Control.

[3] Ng N, Winkler V, Van Minh H, Tesfaye F, Wall S, Becher H (2009). Predicting lung cancer death in Africa and Asia: differences with WHO estimates. Cancer Causes Control.

[4] Peto R, Chen Z-M, Boreham J (2009). Tobacco: the growing epidemic in China. CVD Prev Control.

[5] Andreeva TI, Krasovsky KS (2007). Changes in smoking prevalence in Ukraine in 2001-5. Tob Control.

[6] Rani M, Bonu S, Jha P, Nguyen SN, Jamjoum L (2003). Tobacco use in India: prevalence and predictors of smoking and chewing in a national cross sectional household survey. Tob Control.

[7] Max W, Sung HY, Tucker LY, Stark B (2010). The disproportionate cost of smoking for African Americans in California. Am J Public Health.

[8] Bernat DH, Lazovich D, Forster JL, Oakes JM, Chen V (2009). Area-level variation in adolescent smoking. Prev Chronic Dis.

[9] Hammond D (2005). Smoking behaviour among young adults: beyond youth prevention. Tob Control.

[10] (2009). Ministry of Health, New Zealand.

[11] Stead L, Perera R, Bullen C, Mant D, Lancaster T (2008). Nicotine replacement therapy for smoking cessation. Cochrane Database Syst Rev.

[12] (2009). Ministry of Health, New Zealand.

[13] (2009). Blumberg S, Luke J.

[14] Statistics New Zealand.

[15] Vitalwave Consulting (2009). Washington DC and Berkshire UK, UN Foundation Vodafone Foundation Partnership.

[16] Department of Economic and Social Affairs (2008). United Nations.

[17] Koivusilta LK, Lintonen TP, Rimpelä AH (2007). Orientations in adolescent use of information and communication technology: a digital divide by sociodemographic background, educational career, and health. Scand J Public Health.

[18] Lajunen HR, Keski-Rahkonen A, Pulkkinen L, Rose RJ, Rissanen A, Kaprio J (2007). Are computer and cell phone use associated with body mass index and overweight? A population study among twin adolescents. BMC Public Health.

[19] Rodgers A, Corbett T, Bramley D, Riddell T, Wills M, Lin RB, Jones M (2005). Do u smoke after txt? Results of a randomised trial of smoking cessation using mobile phone text messaging. Tob Control.

[20] Milne K, Bowler S, Li J (2009). The Quit Group.

[21] Whittaker R, Maddison R, McRobbie H, Bullen C, Denny S, Dorey E, Ellis-Pegler M, van Rooyen J, Rodgers A (2008). A multimedia mobile phone-based youth smoking cessation intervention: findings from content development and piloting studies. J Med Internet Res.

[22] Bandura A (1986). Self-Efficacy Mechanism in Psychological Activation and Health Promoting Behavior.

[23] Brennan AJ, Galli N (1985). Health educators: role modeling and smoking behavior. J Drug Educ.

[24] Maddison R, Prapavessis H, Clatworthy M (2006). Modeling and rehabilitation following anterior cruciate ligament reconstruction. Ann Behav Med.

[25] Thelen MH, Fry RA, Fehrenbach PA, Frautschi NM (1979). Therapeutic videotape and film modeling: a review. Psychol Bull.

[26] West R, Hajek P, Stead L, Stapleton J (2005). Outcome criteria in smoking cessation trials: proposal for a common standard. Addiction.

[27] Cooke F, Bullen C, Whittaker R, McRobbie H, Chen MH, Walker N (2008). Diagnostic accuracy of NicAlert cotinine test strips in saliva for verifying smoking status. Nicotine Tob Res.

[28] Wheeler KC, Fletcher KE, Wellman RJ, Difranza JR (2004). Screening adolescents for nicotine dependence: the Hooked On Nicotine Checklist. J Adolesc Health.

[29] Webb TL, Joseph J, Yardley L, Michie S (2010). Using the internet to promote health behavior change: a systematic review and meta-analysis of the impact of theoretical basis, use of behavior change techniques, and mode of delivery on efficacy. J Med Internet Res.

[30] Grimshaw G, Stanton A, Blackburn C, Andrews K, Grimshaw C, Vinogradova Y, Robertson W (2003). Patterns of smoking, quit attempts and services for a cohort of 15- to 19-year-olds. Child Care Health Dev.

[31] Kishchuk N, Tremblay M, Lapierre J, Heneman B, O'Loughlin J (2004). Qualitative investigation of young smokers' and ex-smokers' views on smoking cessation methods. Nicotine Tob Res.

[32] Balch GI (1998). Exploring perceptions of smoking cessation among high school smokers: input and feedback from focus groups. Prev Med.

[33] Grimshaw G, Stanton A (2006). Tobacco cessation interventions for young people. Cochrane Database Syst Rev.

[34] Lipkus IM, McBride CM, Pollak KI, Schwartz-Bloom RD, Tilson E, Bloom PN (2004). A randomized trial comparing the effects of self-help materials and proactive telephone counseling on teen smoking cessation. Health Psychol.

[35] Patten CA, Croghan IT, Meis TM, Decker PA, Pingree S, Colligan RC, Dornelas EA, Offord KP, Boberg EW, Baumberger RK, Hurt RD, Gustafson DH (2006). Randomized clinical trial of an Internet-based versus brief office intervention for adolescent smoking cessation. Patient Educ Couns.

[36] Gnich W, Sheehy C, Amos A, Bitel M, Platt S (2008). A Scotland-wide pilot programme of smoking cessation services for young people: process and outcome evaluation. Addiction.

[37] McCormick LK, Crawford M, Anderson RH, Gittelsohn J, Kingsley B, Upson D (1999). Recruiting adolescents into qualitative tobacco research studies: experiences and lessons learned. J Sch Health.

[38] Martinson BC, Lazovich D, Lando HA, Perry CL, McGovern PG, Boyle RG (2000). Effectiveness of monetary incentives for recruiting adolescents to an intervention trial to reduce smoking. Prev Med.

[39] Menon-Johansson AS, McNaught F, Mandalia S, Sullivan AK (2006). Texting decreases the time to treatment for genital Chlamydia trachomatis infection. Sex Transm Infect.

[40] Puccio JA, Belzer M, Olson J, Martinez M, Salata C, Tucker D, Tanaka D (2006). The use of cell phone reminder calls for assisting HIV-infected adolescents and young adults to adhere to highly active antiretroviral therapy: a pilot study. AIDS Patient Care STDS.

[41] Kim HS, Kim NC, Ahn SH (2006). Impact of a nurse short message service intervention for patients with diabetes. J Nurs Care Qual.

[42] Ferrer-Roca O, Cárdenas A, Diaz-Cardama A, Pulido P (2004). Mobile phone text messaging in the management of diabetes. J Telemed Telecare.

[43] Downer SR, Meara JG, Da Costa AC, Sethuraman K (2006). SMS text messaging improves outpatient attendance. Aust Health Rev.

[44] Whittaker R, Smith M (2008). m-Health: using mobile phones for healthy behaviour change. Int J Mobile Marketing.

[45] Whittaker R, Borland R, Bullen C, Lin RB, McRobbie H, Rodgers A (2009). Mobile phone-based interventions for smoking cessation. Cochrane Database Syst Rev.

